# In Vitro Gene Therapy Using Human iPS-Derived Mesoangioblast-Like Cells (HIDEMs) Combined with Microdystrophin (*μDys*) Expression as the New Strategy for Duchenne Muscular Dystrophy (DMD) Experimental Treatment

**DOI:** 10.3390/ijms252211869

**Published:** 2024-11-05

**Authors:** Marta Budzińska, Agnieszka Malcher, Agnieszka Zimna, Maciej Kurpisz

**Affiliations:** Institute of Human Genetics Polish Academy of Sciences, 60-479 Poznan, Polandagnieszka.malcher@igcz.poznan.pl (A.M.); agnieszka.zimna@igcz.poznan.pl (A.Z.)

**Keywords:** muscular dystrophy, microdystrophin, gene therapy, *mdx* mice, Duchenne Muscular Dystrophy

## Abstract

Duchenne Muscular Dystrophy (DMD) is a genetic disorder characterized by disruptions in the dystrophin gene. This study aims to investigate potential a therapeutic approach using genetically modified human iPS-derived mesoangioblast-like cells (HIDEMs) in *mdx* mouse model. This study utilizes patient-specific myoblasts reprogrammed to human induced pluripotent stem cells (iPSCs) and then differentiated into HIDEMs. Lentiviral vectors carrying microdystrophin sequences have been employed to deliver the genetic construct to express a shortened, functional dystrophin protein in HIDEMs. The study indicated significant changes within redox potential between healthy and pathological HIDEM cells derived from DMD patients studied by catalase and superoxide dismutase activities. Microdystrophin expressing HIDEMs also improved expression of genes involved in STARS (striated muscle activator of Rho signaling) pathway albeit in selective DMD patients (with mild phenotype). Although in vivo observations did not bring progress in the mobility of *mdx* mice with HIDEMs, microdystrophin interventions this may argue against “treadmill test” as suitable for assessment of *mdx* mice recovery. Low-level signaling of the Rho pathway and inflammation-related factors in DMD myogenic cells can also contribute to the lack of success in a functional study. Overall, this research contributes to the understanding of DMD pathogenesis and provides insights into potential novel therapeutic strategy, highlighting the importance of personalized gene therapy.

## 1. Introduction

Duchenne Muscular Dystrophy (DMD) is an X-linked, recessive disorder affecting 1 in 5000 male live births [1]. Disruptions in open-reading frame in dystrophin gene (*DMD*) are caused by deletions (roughly 65%), duplications (10%), point mutations (10%), or other smaller rearrangements [2]. Those genetic rearrangements may lead to the loss of dystrophin protein, which has a crucial impact on striated muscle cells. Differences in *DMD* mutations are responsible for different DMD patient’s phenotype. In Becker muscular dystrophy (BMD), the truncated version of dystrophin is being produced, which causes milder symptoms and achieves a longer lifespan in Becker individuals than those with DMD. The distinction between DMD and BMD is mostly explained by the differences in the reading frame rule. In DMD we can observe mostly frame-disrupting or out-of-frame mutations, which lead to the lack of translation, while in BMD we can observe the deletion of an exon, without reading frame alteration which leads to the production of a truncated version of dystrophin. The studies suggest that increase of dystrophin expression (>4%) may benefit the general muscle vitality [3]. This information led to an investigation on exon skipping and patient-specific cell approaches in DMD cellular therapies [4,5]. In our studies, we used DMD patients with different phenotypes of whose cells were reprogrammed to patient-specific human iPS cell-derived mesoangioblast-like cells (HIDEMs). This cell type may be used as the precursor of myocytes using the Maffioletti protocol [6] and then genetically modified for the expression of microdystrophin.

One of the important factors accompanying DMD is the destabilization of the redox environment in myocytes. Contractile activity of myocytes results in an increased production of ROS and other free radicals, promoting a pro-inflammatory environment [7]. It has been shown that DMD patients and *mdx* mice can manifest altered enzymatic antioxidant responses, increased protein oxidation, and enhanced levels of oxidized glutathione [8,9,10]. Those examples justify the need for additional study on ROS and their connections with muscle differentiation and physiology, for a better understanding of the DMD pathogenesis. Normally, inflammation-derived ROS play a different roles in the process of muscle repair: on the one hand, ROS participate in many events supporting muscle repair, while on the other hand, persistent production of ROS by infiltrated neutrophils leads to cell injury [11,12]. Such ambiguous action may be modified by different transcription factors and signaling pathways. Studies on antioxidant treatment in *mdx* mice revealed the prevention of muscle waste by decreasing of oxidative stress and inhibition of NF-κB transcription factor [11].

Another findings were related to the striated muscle activator of Rho signaling (STARS), as a muscle-specific protein, which has been proposed to play an important role in skeletal muscle functions, and regulation of MRFT-A/SRF axis [13]. Serum response factor (SRF) is a ubiquitously expressed and highly conserved downstream signaling target of the Rho signaling pathway and has been linked with skeletal muscle remodeling and development, [14] and plays an important role as the regulator of genes responsible for cell growth and differentiation thus inducing myogenesis [15]. SRF is activated through signaling of Rho GTPases and actin filament dynamics [16,17]. It was identified as an actin-interacting protein and myocardin-related transcription factor A (MRTF-A), as a crucial coactivator of SRF [18]. Transcription of Rho-dependent MRTF/SRF can be activated by any extracellular signal, which leads to actin polymerization. Genes with SRF binding sites include genes that are important for music cells stability such as: dystrophin, alfa-actin, and tropomyosin gene [15]. Pharmacologic disturbances of MRTF-A/SRF result in elevated myofibroblast apoptosis [19]. This information supports that the Rho/Actin/MRTF/SRF signaling axis is an important intersection point for profibrotic signals leading to myofibroblast differentiation or tissue fibrosis that could be expected for DMD patient’s benefit [19,20].

It is well-known that *DMD* is the largest known human gene, and its size increases vulnerability to different genetic rearrangements. As a consequence, the efficient therapeutic approach ideally should be universal. In our studies we combined the lentiviral vector with microdystrophin (*μDys*) sequence to provide the production of shortened, but functional dystrophin protein by Maffioletti protocol [6] to provide genetically modified HIDEMs that could be an option for HIDEMs cellular therapy. Additionally, we examined the redox environment and the expression levels of STARS pathway members as important factors accompanying DMD that could be beneficially changed by proposed gene therapy. Finally, we attempted to test functionally obtained biological medicine (HIDEMs–microdystrophin transfected) with *mdx* mice imitating congenital muscular dystrophy.

## 2. Results

### 2.1. iPS Cells In Vitro Characteristics

**Figure 1:**
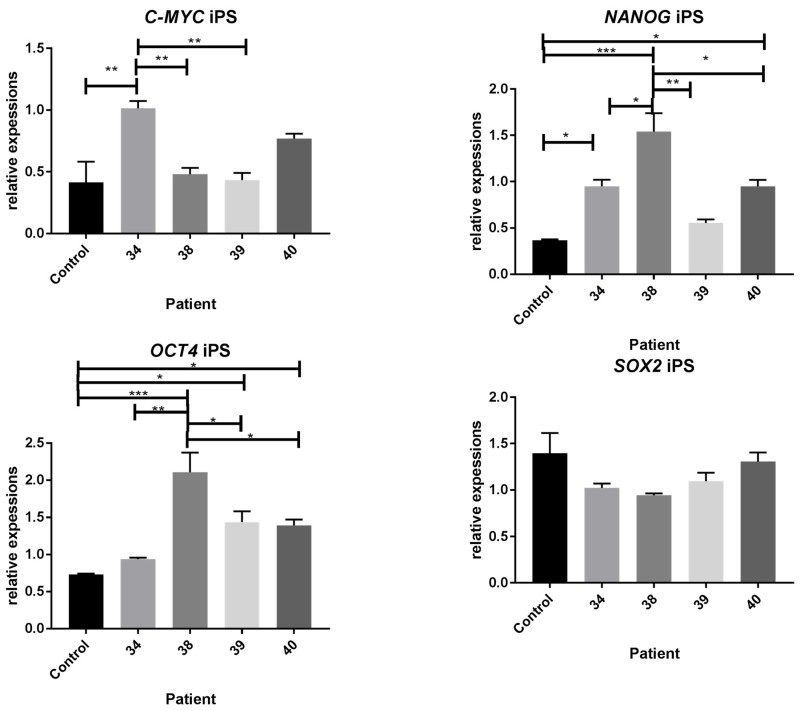
The expression level of pluripotency marker genes (*C-MYC*, *NANOG*, *OCT4*, *SOX2*) in control and DMD patients’ iPS cells. Reference genes: *ACT*, *TBP*, *GAPDH*. Statistical significance has been shown by *p* < 0.05 (*), *p* < 0.01 (**), *p* < 0.001 (***). Results were performed using the one-way ANOVA test.

Results were obtained as relative gene expression (of pluripotency genes) (Figure 1) and confirmed the pluripotent character of DMD patients-derived iPS cells. The iPS from patient 38 had the highest relative expression level of *OCT4* and *NANOG* (*p* < 0.001) compared with control. In cells derived from patient 34, we observed statistically significant increased expression of *C-MYC* and *NANOG* (*p* < 0.01), while in patient 40 were significantly increased *NANOG* and *OCT4* mRNA levels (*p* < 0.05). In patient 39 we have observed significantly increased expression level of *OCT4* (*p* < 0.05). In all the patients’ cells revealed similar non-significantly increased *SOX2* relative expression.

**Figure 2:**
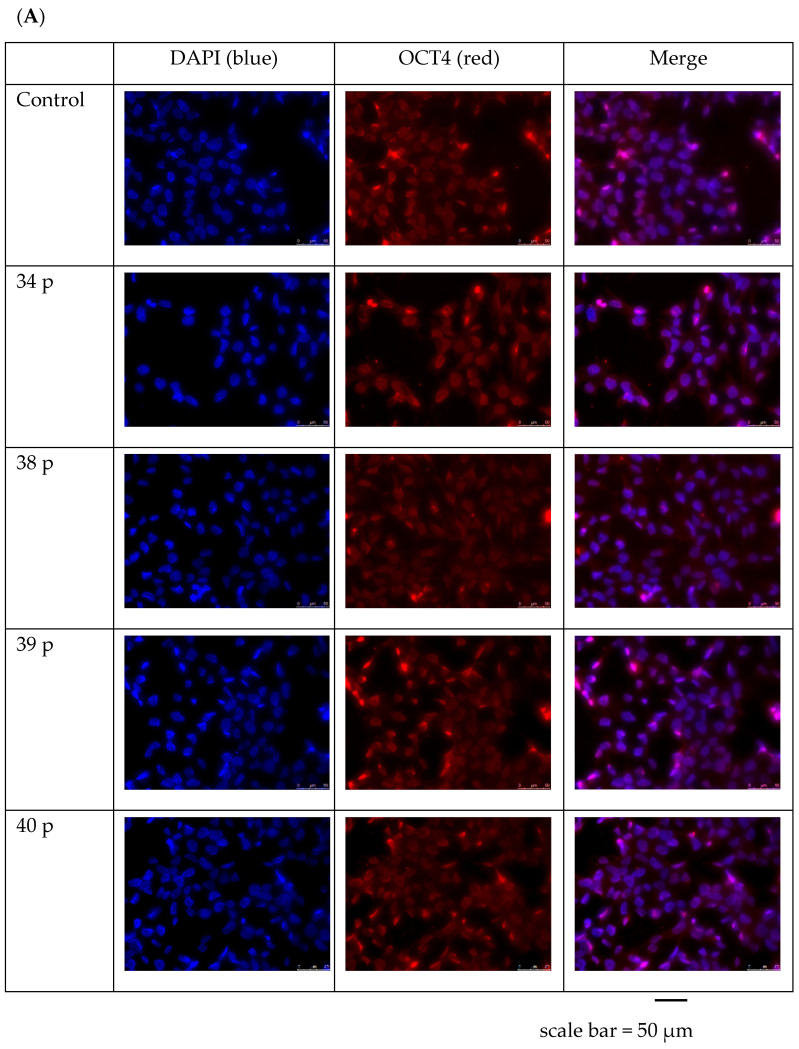

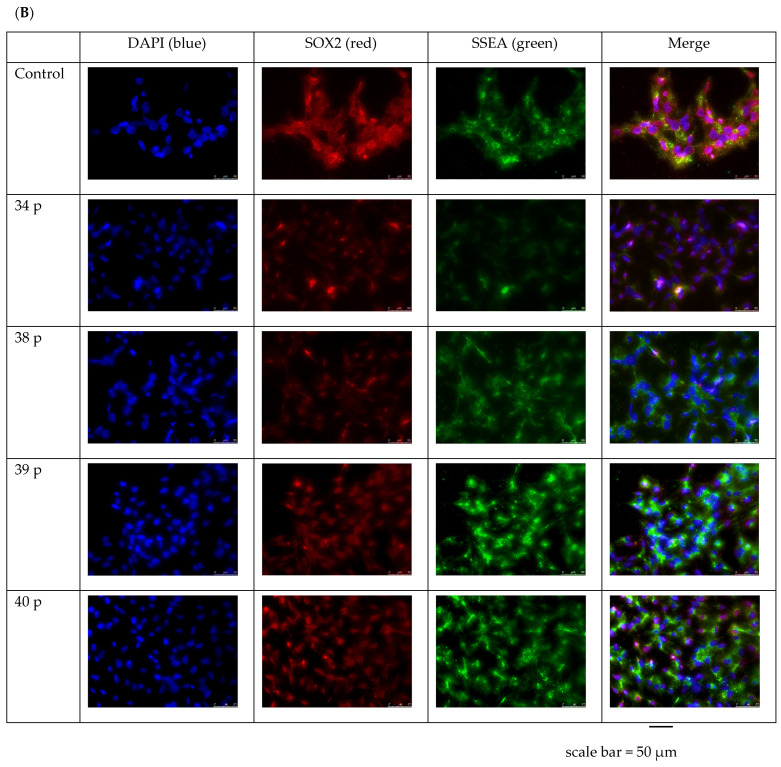

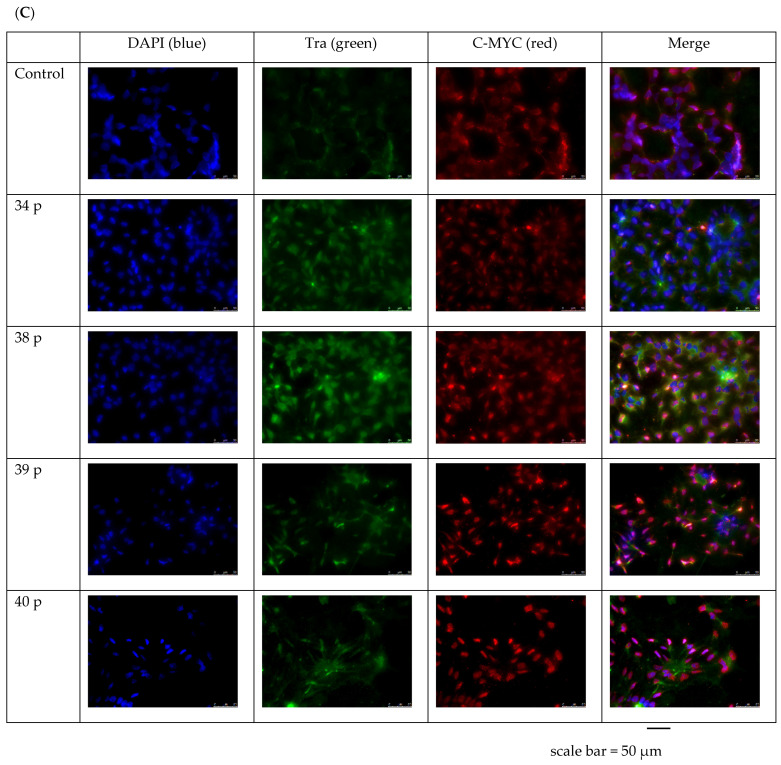

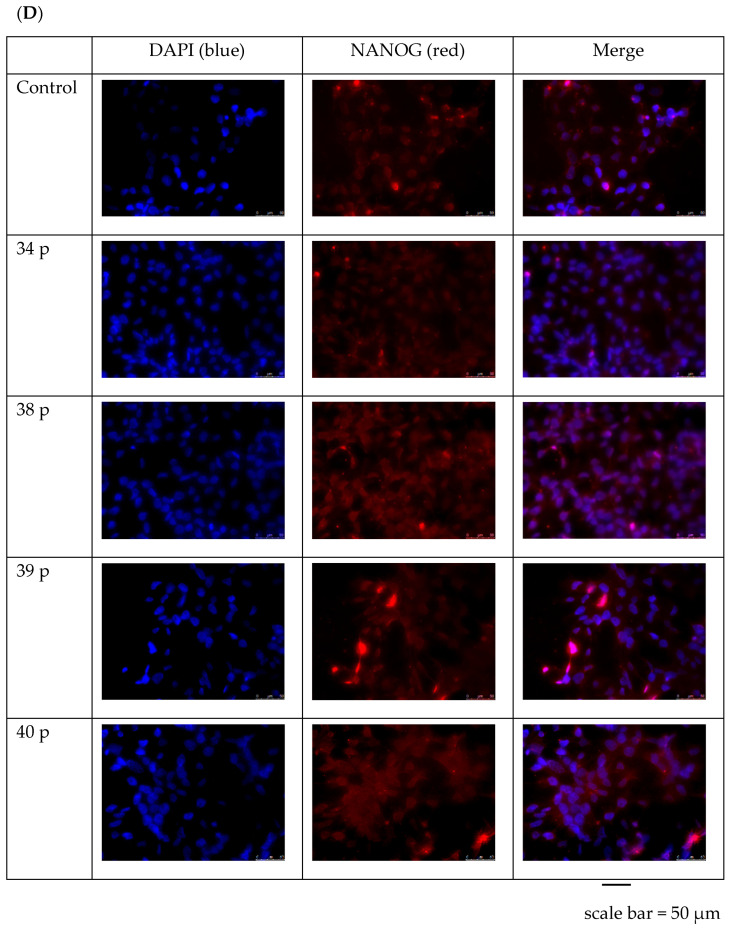
The immunofluorescent staining of OCT4, SOX2, SSEA, NANOG pluripotency markers in control and DMD patients iPS cells. Cells were investigated at the 10th in vitro cell culture passage. Identical patients’ samples were investigated for (**A**): OCT4, (**B**): SOX2, (**C**): CD 73 and CD 31, (**D**): CD 13 biomarkers. The images were obtained using Leica DMi 8 microscope (Leica Microsystems, Wetzlar, Germany), with 40× zoom.

Those characteristics were also visible in immunofluorescent staining of studied in vitro-cultured iPS cells (Figure 2). The cells staining revealed some differences in the pluripotency markers expression between patient samples under study. For NANOG staining, the highest fluorescence was confirmed in patient 38 cells. The insensitivity of fluorescence for SOX2 was similar among all the patients’ cells studied, while SSEA staining was the most intensive in 39 and 40 patients’ cells. The C-MYC and NANOG signals were the most intensive in 38 patient’s cells.

### 2.2. Differentiation of HIDEMs

**Figure 3:**
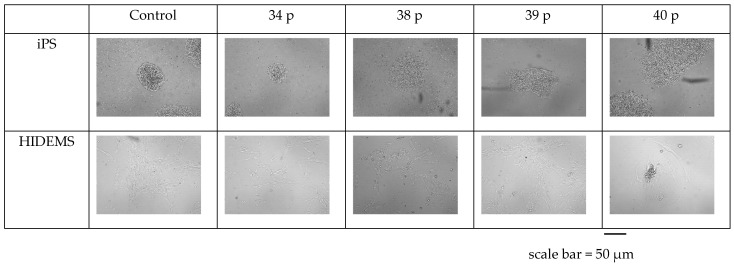
The differences in morphology between iPS and HIDEM cells. iPS cells were observed at the 10th in vitro cell culture passage (6 well Tissue Culture Well, Falcon), and HIDEMs at the 2nd in vitro passage (75 cm^2^ Cell Culture Flask, Falcon). Images were captured using JuLI FL analyzer (NanoEntek, Seul, Republic of Korea) in 4× zoom.

During the differentiation process, the changes in the size of cell colonies and their morphology were observed (Figure 3). At the beginning, the iPS cells were agregated, with dense cytoplasm and centrally located nuclei. During the differentiation, the cytoplasm elongated and the nuclei were decentralized. The process was consistent with that one reported by Maffioletti et al. [6]. The patient No 40 derived HIDEM cells were considered as the most problematic ones. They had significantly more cytoplasm and more round shape than the other patients and control cells. We undertook three attempts (for cells derived out of patient 40) of differentiation into HIDEMs, after the 2nd in vitro passage and we always observed the lack of further proliferation which in consequence ended up with limited characterization of those cells.

### 2.3. HIDEM Cells Characteristics

**Figure 4:**
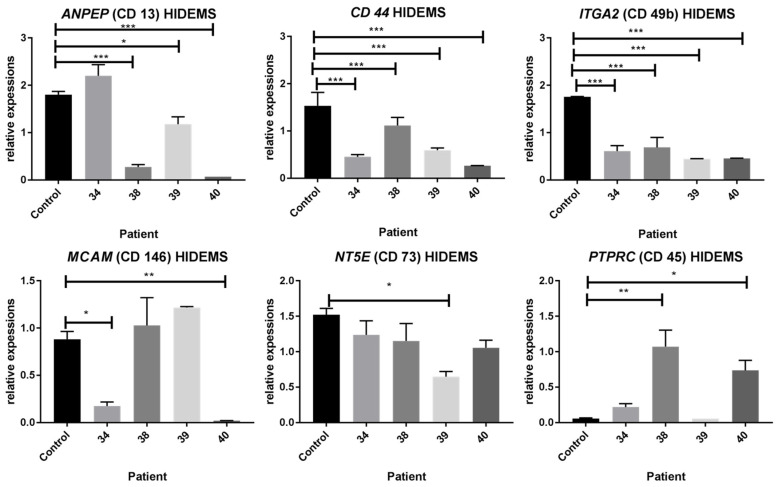
The expression level of mesenchymal marker genes: *ANPEP* (CD 13), *CD 44* (CD 44), *ITGA2* (CD 49b), *MCAM* (CD 146), *NT5E* (CD 73) and *PTPRC* (CD 45) genes in control and DMD patients’ derived HIDEMs. Reference genes: *ACT*, *HPRT*, *GAPDH*. Statistical significance has been shown: *p* < 0.05 (*), *p* < 0.01 (**), *p* < 0.001 (***). Results were assessed using the one-way ANOVA test.

The expression level of HIDEMs marker genes was different among analyzed DMD patients and totally different from the cells derived from control individual. Results were next confirmed by immunostaining and flow cytometry indicating the diversified character of cells obtained. HIDEMs derived from patient 38 revealed the most similar expression level to mesenchymal markers when compared with the healthy control cells (Figure 4). The patient 40 HIDEMs represented the most pronounced differences within examined markers expression levels.

**Figure 5:**
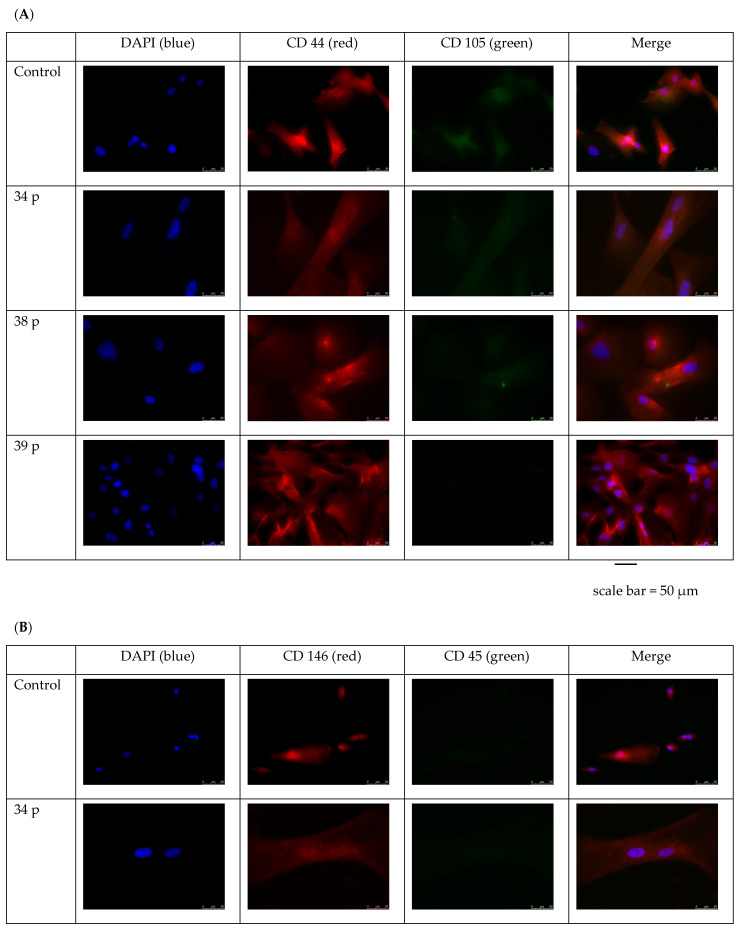

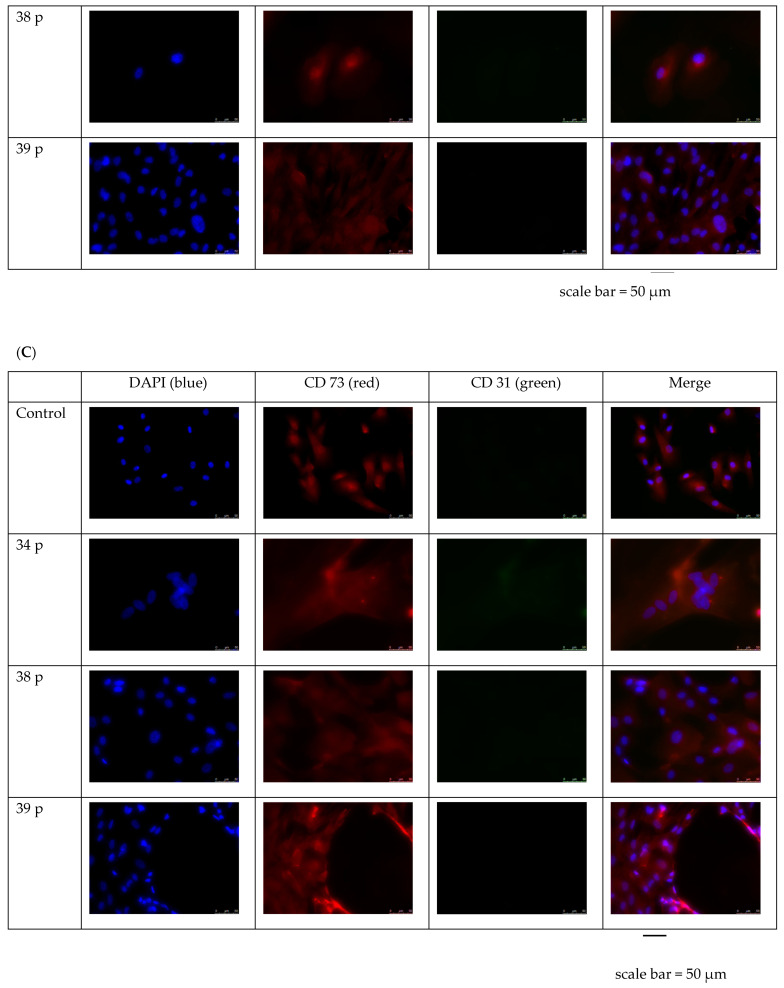

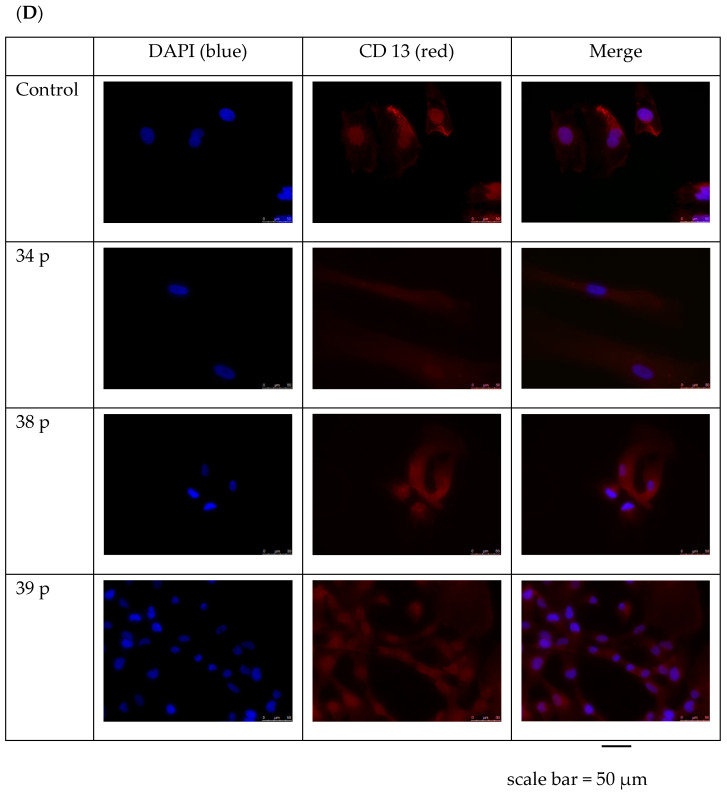
The immunofluorescent staining of mesenchymal cell markers (CD 44, CD 45, CD 146, CD 45, CD 73, CD 31, CD 13, and CD 105) in control and DMD patient’s derived HIDEMs. Cells were investigated at the 3rd in vitro cell culture passage. Identical patients’ samples were investigated for (**A**): CD 44 and CD 105, (**B**): CD 146 and CD45, (**C**): CD 73 and CD 31, (**D**): CD 13 markers. The images were obtained using Leica DMi 8 microscope (Leica Microsystems, Wetzlar, Germany), with 40× zoom.

The presence of the marker proteins was investigated using immunofluorescent staining which confirmed the mesenchymal character of the cells obtained (Figure 5). The results from patient 40 were not available due to the lack of their proliferation. Generally, the cells obtained from patients 34, 38 and 39 were CD 44, CD 146, CD 73, and CD 13 positive, however, the weak signals of fluorescence were noted in the case of cells from 34 and 38 patients, which were similar to the cells out of the control. CD 45, CD 31, and CD 105 staining were very weak in all investigated HIDEM cell suspensions.

**Figure 6:**
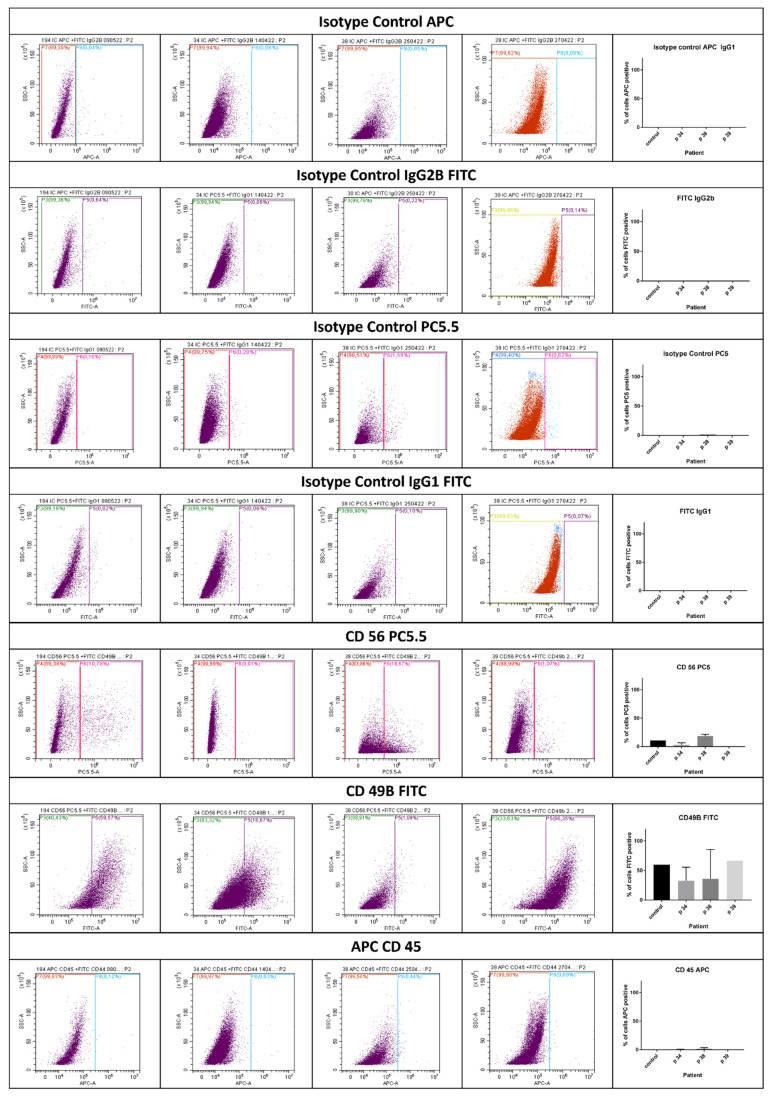

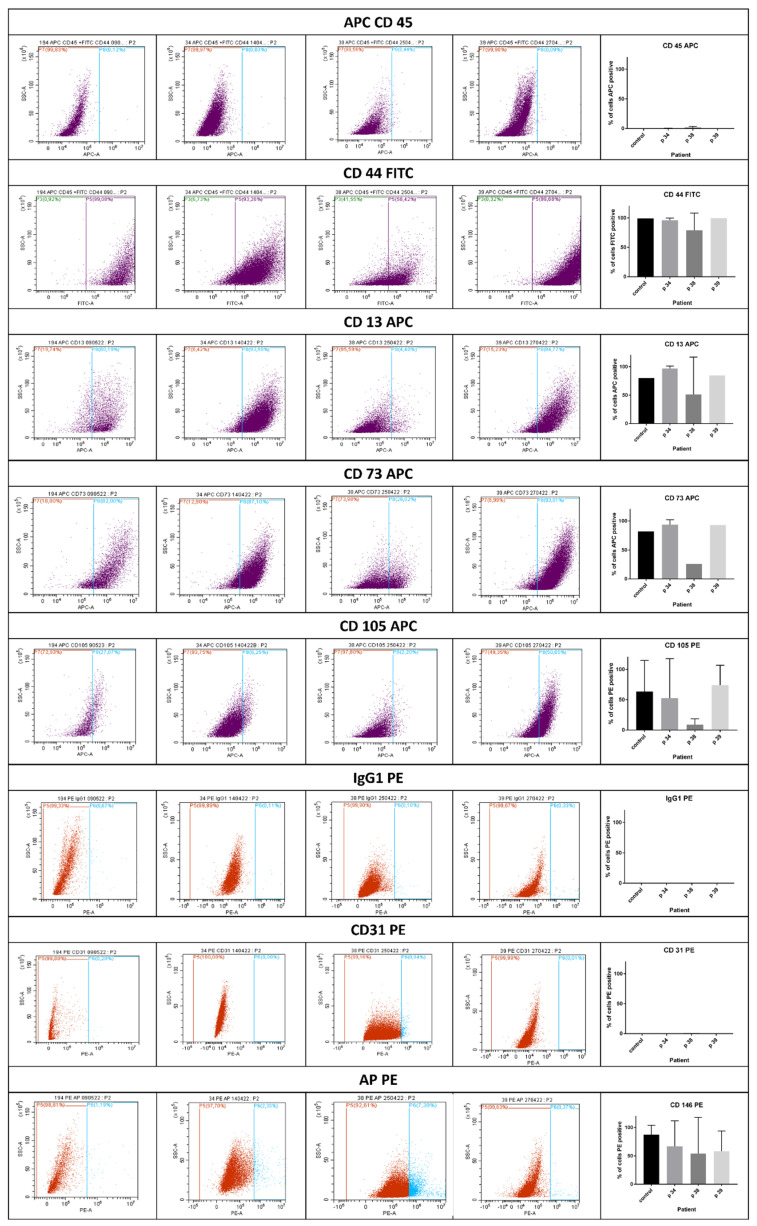
The flow cytometry staining for mesenchymal markers in control and HIDEMs cell populations.

HIDEMs were also investigated using flow cytometry (Figure 6). Cells were CD 49b, CD 44, CD 13, CD 73, CD 105, and alkaline phosphatase (AP) positive. The significant differences between the cell samples under study were not observed. All of the patients’ HIDEMs presented the phenotype which was similar to that one observed in the cells derived from the control individual. However, the expression of marker antigens varied among the different patients’ cell populations, for example, patient 38 demonstrated rather low weak staining for CD 105 and CD 73. The results were obtained in two technical replicates.

### 2.4. HIDEMs Lentiviral Transduction

**Figure 7:**
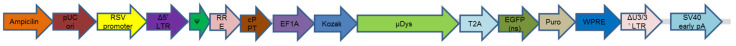
Lentiviral l vector expressing the microdystrophin (*μDys*) sequence.

**Figure 8:**
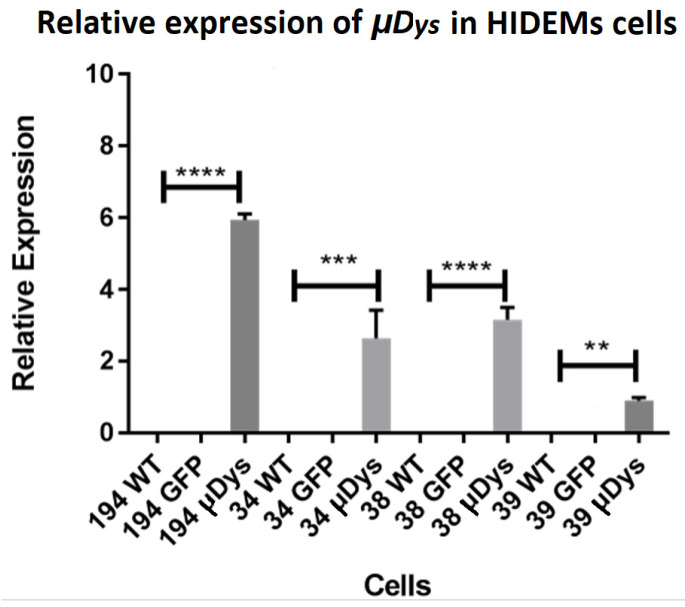
The expression level of mRNA transcripts of *μDys* before and after genetic modification of HIDEM cells. All transduced cells showed a significant relative expression of *μDys* with respect to non-transduced cells. Statistical significance has been shown: *p* < 0.01 (**), *p* < 0.001 (***), *p* < 0.0001 (****). Results were performed using the one-way ANOVA test.

**Figure 9:**
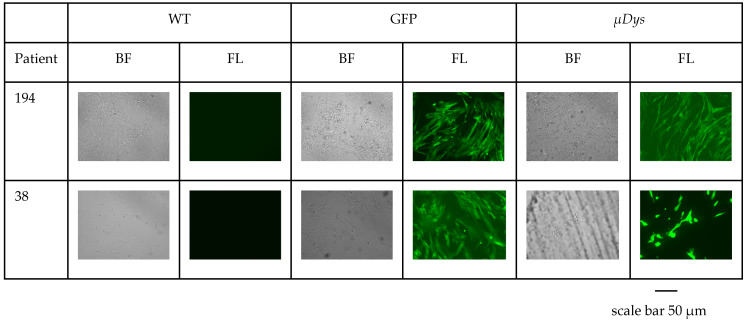
The overview of transduced HIDEM cells. HIDEM cells were observed on the 7th day of in vitro culture after transduction (100 × 20 mm Tissue Culture Dish, Falcon). Images were captured using JuLI FL analyzer (NanoEntek, Seul, Republic of Korea) in 40× zoom. Visible fluorescence of cells confirms the efficient transduction process. Abbreviations: WT—wild type, GFP—Green fluorescent protein transduction, *μDys*—microdystrophin transduction, BF—bright field canal, FL—fluorescence canal.

The relative expression of *μDys* has confirmed the lentiviral intervention (a scheme for lentiviral vector has been presented in Figure 7) success in all cell populations derived, with the best results for patient 38 cells (*p* < 0.0001), which revealed a 4-fold increased relative expression of *μDys* and with an exception of patient 40 (Figure 8). (In control cells, the relative expression of microdystrophin was 6-fold increased). Patient’s 40 HIDEM cells stopped the cell proliferation, which made them unable to be subjected to transduction. The best reactive cell population derived from patient 38 presented good morphology as it has been shown in Figure 9. 

### 2.5. Redox Potential of HIDEM Cell Homogenates

**Figure 10:**
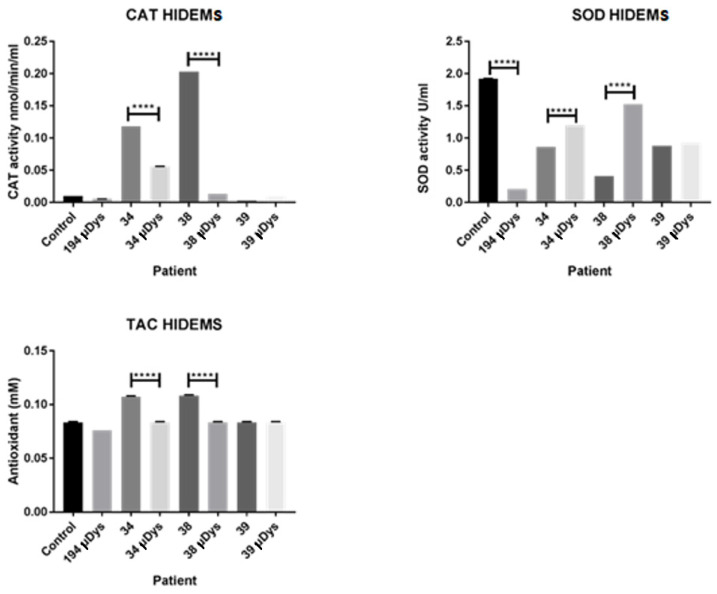
The level of CAT SOD and TAC activities before and 2 weeks after genetic modification of HIDEM cells, *p* < 0.0001 (****).

In general, CAT activity and TAC were decreased in HIDEM cells after the microdystrophin transduction. CAT activity was significantly lower (*p* < 0.0001) in lysates out of cells from patients 34 to 38 compared to the cells without transduction (Figure 10). The total antioxidant capacity was also decreased (*p* < 0.0001) in all lysates from cell suspensions of patients 34 and 38 comparing to control cells without transduction. The cells from patient 39 did not reach the statistical differences before-and-after transduction, however the cells demonstrated almost no CAT activity. We have also observed the increased activity of SOD in the lysates of HIDEM transduced cells derived out of DMD patients 34 and 38.

**Figure 11:**
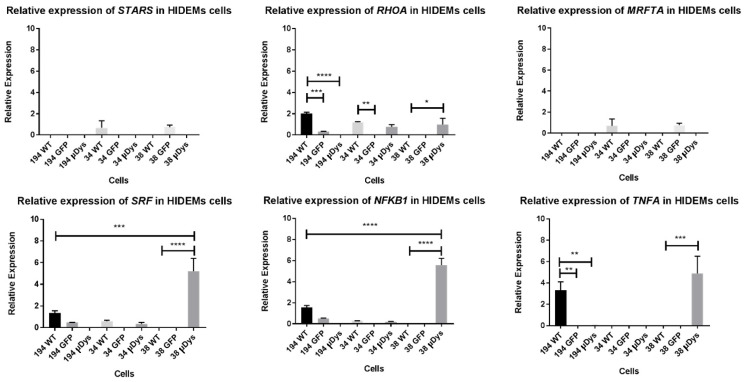
The comparison of the expression level of STARS pathway members in HIDEMs cells before and after lentiviral transduction. Statistical significance has been shown: *p* < 0.05 (*), *p* < 0.01 (**), *p* < 0.001 (***), *p* < 0.0001 (****). Results were assessed using the one-way ANOVA test.

Relative expression levels of STARS pathway members showed differences among patient’s phenotypes (Figure 11). Investigation on primary triggered gene (*STAR*) did not show any specific changes in expression level before-and after transduction of HIDEMs. We have observed, however, increased level of *SRF* expression in cells from patient 38 (*p* < 0.0001). Moreover, increased expression level for *NFKB1* (*p* < 0.0001) and *TNFA* (*p* < 0.001) genes were observed in the same, 38 patient’s transduced cells (homogenate).

### 2.6. Animal Studies

**Figure 12:**
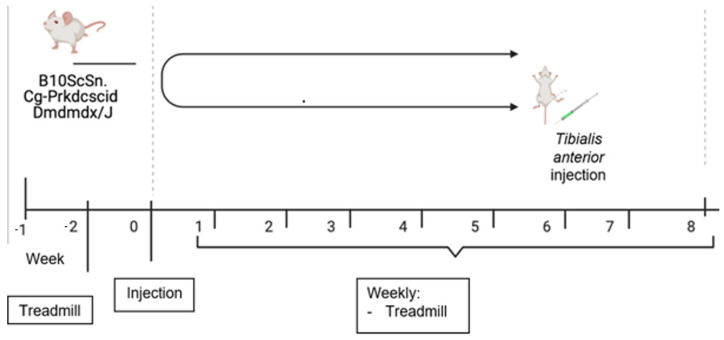
Animal experimental scheme.

After obtaining the results from the biological characterization of HIDEM cells from individual DMD patients, i.e., analyzing the results of gene expression levels for the STARS signaling pathway and the activity of antioxidant enzymes, HIDEMs from patient 38 were selected for transplantation into hind limb muscles in *mdx* mice of different subgroup (Figure 12) according to protocol by Gerli et al. [21].

**Figure 13:**
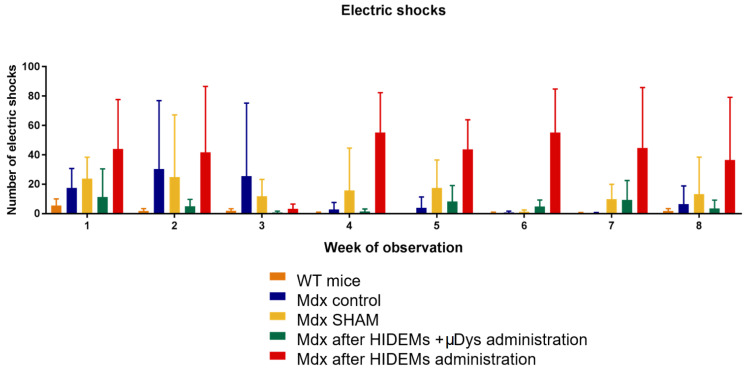
Average number of electric shocks exerted to *mdx* mouse groups under study.

**Figure 14:**
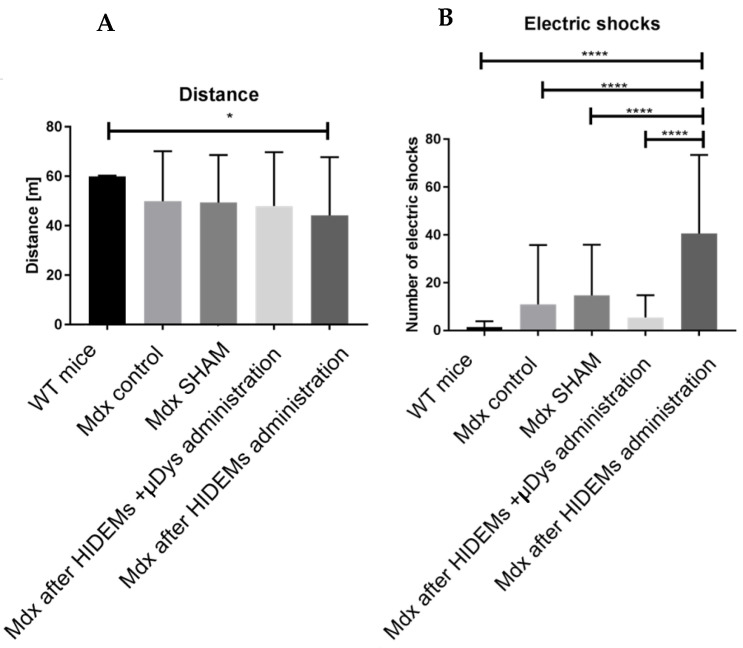
Diagrammatic representation of the entire 8-weeks observation of the treadmill exercise in *mdx* mouse groups (**A**): The sum of traveled distance in mouse groups. (**B**): Average number of electrocutions (electric shocks) inflicted to test mouse groups; *p* levels were as follows: * *p* < 0.05, **** *p* < 0.0001.

Behavioral observations of *mdx* mice revealed significant individual differences in the length of reached distance. The minimum distance between the test groups *mdx* was 2 m, while healthy mice (WT) showed a minimum distance travelled of 58 m. The median obtained for the test subgroups of *mdx* mice was similar. The standard deviation (+/−) for the respective test *mdx* groups was the following: 21.32; 19.45; 21.85; 23.61 (Table 1) and differed significantly from that one obtained for the WT mouse group (0.37). Some differences in distance were, however, accompanied by differences in the number of electric shocks (Table 2, Figure 13). A minimum of 12 episodes were observed in the WT group of mice, while individuals in the *mdx* group of ‘native’ pathological mice received up to 100 shocks in which warning signal has been interpreted as a threat to the animal and in fact terminated the experiment. In a summary, the travelled distance (with exception of control mice) did not differ significantly (Figure 14A) while the number of electrocutions reached statistical significance in studied subgroups (Figure 14B). 

There was a visible trend for reduction in the number of electrical shocks in *mdx* mice after intervention with HIDEMs + μ*DYS* as compared to the other test groups of *mdx* mice from the treadmill exercise of which value was highly statistically significant (Figure 14; *p* < 0.0001).

## 3. Discussion

DMD has been a subject of research both fundamentally and clinically. After many attempts, at present, treatment of DMD has been focused on manifested symptoms instead of its reason.

One of the proposed therapies for underlined genetic background was the so-called exon skipping. Komaki et al. proposed systemic administration of the antisense oligonucleotide NS-065/NCNP-01 for skipping exon 53 in Japanese boys with a confirmed diagnosis of DMD, within a reading frame that could be then restored in DMD gene. For all selected patients, it was induced the expression of dystrophin mRNA (which was slightly shorter than wild-type dystrophin) [5]. However, this approach was not suitable for all the DMD patients due to the specific construction of antisense oligonucleotide. It was rather understood the further need for more universal approach which could enable a successful therapy for the whole DMD patients spectrum.

**Figure 15:**
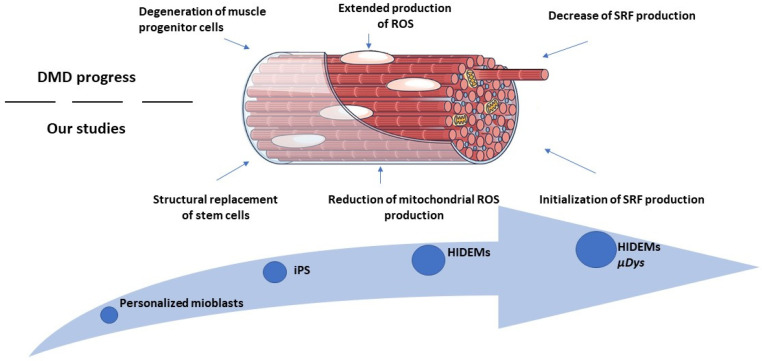
Elements of HIDEMs therapeutic interactions with mechanisms affecting skeletal muscle dysfunction in course of DMD.

In our studies we used the DMD patients’ myoblast cells to obtain iPSc from which we have differentiated HIDEM cell precursors. Then, we performed lentiviral transduction with microdystrophin sequence to obtain genetically correct, patient-specific cells. This approach can give the opportunity for personalized treatment, without dangerous and persistent reactions of the immune system. Genetically corrected HIDEMs may interact with dystrophic muscles in many ways which can be illustrated in Figure 15 that, in part, demonstrates the results obtained in our study. In our view, the studies on the administration of cells with microdystrophin sequence using viral vectors seem to be the most promising approach. Potter et al. had performed a pre-trial with systemic injections of rAAVrh74.MHCK7.micro-dystrophin construct in *mdx* mice as a sort of preclinical study [22]. However, our approach seems to be more consistent with Mendell et al. studies [23] in which they performed an intravenous injection to deliver the AAV construct containing *μDys* to DMD individuals. Four patients had confirmed the stability of transduction and showed functional improvement of muscles and reduced creatine kinase levels, that were maintained for 1 year. The latter trial indicated positive results of novel medical intervention, which can be well tolerated and have minimal adverse effects.

In our study, we used specific, patient-derived HIDEM cells that were genetically modified in order to correct muscle cells useful for prospective cell therapy. The main aim of this approach was to recuperate the loss of the affected skeletal muscle tissue, however, similar to myoblast transplantation in patients with muscular dystrophy (MD) [24], we aimed for local administration to locomotoric muscles. Mesoangioblast cells have been considered attractive for cellular treatment as they can be systemically delivered and colonize the skeletal muscles when penetrating the vessels. Cossu et al. performed systemic transplantation of mesoangioblasts, which proved partially efficacious in experimental therapy of DMD [25]. Our results partly support these findings and suggest that a combination of cell and gene therapy may be a right direction for next approaches. However, it seems that the success of such approach may depend on the type of *DMD* progression. In our study, the phenotype was closely related to the cell in vitro culture success. We could not obtain, for example, the cell characteristics of HIDEMs derived from patient 40, which could be explained by the complex mutations in this individual (heavy phenotype, tetraplegia). The patient responded well to the differentiation process to obtain HIDEMs, but at the 2nd in vitro passage, the cell proliferation was inhibited. Dystrophin is the protein which strongly supports the cell membrane and prevents its damage. Also, the differences in HIDEM cells morphology in patient No 40 may be explained by the complexity of the *DMD* mutations which affected the cell cytoskeleton. HIDEMs could not take on the proper cell shape. HIDEM cells are bigger and much more elongated than iPS cells, which makes them more vulnerable to damage.

We also tried to investigate the influence of genetic engineering on the redox environment of obtained cells. We acknowledged upregulated levels of ROS and antioxidants in skeletal muscles of DMD patients and *mdx* mice [8,26,27]. In our studies, we found a tendency which suggests that a lentiviral construct intervention (with microdystrophin) decreased oxidative stress. However, the observed recovery was more efficient in the cells of patients with milder DMD phenotype. In such individuals, we have observed that CAT and TAC levels were reduced compared to the pre-treated cells. Those findings suggest a supportive role of µ*DYS* in the redox environment, which ameliorated ROS production. Moreover, the SOD activity level significantly increased in the same cell homogenizates, which may suggest that transduction of microdystrophin supported mitochondrial activity, of which side effect can be superoxide production, neutralized by SOD.

It has been proved that Rho GTPases are under the regulation of redox environment of the cell [28]. RhoA plays an important role in the activation of several transcription regulators including NF-κB. It has been noted that RhoA activation is linked to increased *c-Myc* expression through NF-κB activation [29,30]. As mentioned earlier, the NF-kB transcription factor is also an important regulator of pro-inflammatory reactions. In our patient 38 derived HIDEM cells transduced with µ*DYS* demonstrated statistically significant higher expression of NF-kB. On the one hand, that was unexpected according to the relation of NF-kB with ROS, on the other hand the NF-kB is under the regulation of RhoA. In light of those results, it may be that triggered antioxidant reactions in DMD cells can inhibit the proinflammatory NF-kB activation pathway since this transcription factor is regulated by RhoA, of which expression was elevated in transduced with microdystrophin cells of patient 38. Interestingly, the TNF-A mRNA expression was also increased, which may seem opposite to the lower antioxidant activity in HIDEM cells after genetic modification. However, the TNF-A was found to be responsible for the stimulation of SRF binding to the serum response element (SRE) and controlling the same element in satellite cells [31]. Our data seem to be consistent with those results.

*SRF* element of STARS signaling pathway was elevated in HIDEM transduced cells of patient 38, which may indicate that µ*DYS* administration supports DMD patient’s cells and may be an interesting target in planned future in vivo studies in dystrophic mice. Sadler et al. observed significantly reduced levels of mRNA and proteins of STARS pathway members in the *tibialis anterior* (TA) muscle from the dystrophin-deficient *mdx* and *quadriceps* muscle from patients with DMD [32]. The overexpression of human STARS member (hSTARS) in the TA muscles of *mdx* mice increased maximal isometric specific force by 13%, with no changes in muscle fibres or morphology. Therefore, STARS may have had a positive impact on *mdx* muscles, supporting muscle function and recovery. Those findings are consistent with our observations, in which we underlined the increased expression of STARS pathway members at mRNA level after the lentiviral transduction with microdystrophin. Moreover, all the mentioned studies confirmed the therapeutic value of STARS pathway regulation in DMD therapy. The increase of the SRF relative expression suggests the additional value of the µ*DYS* administration, which supports the benefits of DMD treatment. Differences in relative expression profiles in investigated patients suggest, that the scale and mechanisms which contribute to DMD gene may be also different.

Important part of our studies was the animal testing. Treadmill test results revealed a tendency for decreased distance for ’native’ *mdx* mice and increased number of shocks during the experiments. The trend for decreased number of electric shocks received by the *mdx* animals which were subjected to HIDEMs + *μDys* cell administration was, however, highly statistically significant (*p* < 0.0001). This can be, in our view, an indirect proof of skeletal muscle recovery since a learning skill of “mdx” mice clearly prevented them in non-treated or native groups from further exercise in treadmill experiments. As we have strictly followed a protocol by Gerli et al. [21] in terms of intramuscular cell transplantation volume and number of HIDEM cells it is highly unlikely that we have applied insufficient number of cells to the site (*tibialis anterior*) although an *mdx* model of general muscular dystrophy cannot be compatible with Geri et al. experiments in which local limb-girdle muscular dystrophy (LGMD mice) has been applied [21]. This could be the possible reason of less spectacular results obtained on our study. In turn, the lack of significant differences in traveled distance may be explained by low number of tested animals or their relative low muscle dysfunction different than can be observed in human DMD (n = 4). Observations of the reached distance in the tested groups revealed also, the influence of behavioral factors on the outcome of the tests. Mice that were repeatedly electrocuted learned the mechanism that accepting a prolonged single shock ended the experiment, so that they could stop to run on the treadmill avoiding repeated electrocution.

Gene construct transfer to the muscle cell precursors (HIDEMs) seems to be the most promising molecular approach for neuromuscular diseases treatment. Quite recently, single-dose delivery of the *SMN* gene (SMN1 AC005031) to infants with spinal muscular atrophy (SMA) type 1 using Zolgensma (adeno-associated virus serotype 9) has been shown to be crucial for patients survival [33,34]. In light of this finding, ongoing research may provide key information for novel successful treatment of DMD regarding genetic correction together with cellular therapy colonizing the whole locomotoric system of skeletal muscles.

## 4. Materials and Methods

### 4.1. Materials

#### 4.1.1. Patients

The group of interest was conducted on four patients with confirmed DMD gene mutations (Table 3). Patients 34 (deletion of exons 8–22) and 38 (deletion of exons 51–57) represented the milder phenotype, while patient 39 in age of 15 (duplication of exons 53–57) and patient 40 (complex deletions) were tetraplegic at the beginning of the study (heavy phenotype).

#### 4.1.2. Animals

The study used B10ScSn.Cg-Prkdc<scid> Dmd<mdx>/J mice purchased from Jackson Laboratory, Bar Harbor, ME, USA. The heterozygotic mice were used in the breeding pairings (The Institute of Human Genetics Polish Academy of Sciences has permission to breed the mdx mice). Eight to ten-week old male *mdx* mice were split into several groups: a control group ‘native’ (n = 4), a group that received HIDEM cells (n = 4), and a group that received injection of physiological saline “SHAM” (n = 4). Another test group was established for those ones that received HIDEM cells transduced with the microdystrophin sequence (*μDys*). The WT (positive) control (n = 4) group was made out of C57BL mice while non-treated *mdx* served as another control (n = 4). Animal studies have received permission No 59/2021 obtained from the Local Ethical Committee at the University of Life Sciences, Poznan, Poland.

### 4.2. Methods

#### 4.2.1. Isolation of Human Myoblasts

Procedures with human cells were held upon Bioethical Commission at Wielkopolska Medical Chamber approval (Resolution No 199/2019 dated on 18 September 2019). Human primary suspensions of myoblasts were isolated from a ~1 cm^3^ fragment of skeletal muscle tissue harvested out of obtained skeletal muscle biopsy from DMD patients (n = 4) in MedPolonia Hospital, Poznan. Briefly, the biological samples were stripped off the fat and after three digestions with 0.2% collagenase 1 solution (Sigma-Aldrich, Darmstadt, Germany) in a water bath (37 °C), the cell suspensions were filtered through 70 μm mesh. Finally, cells were centrifuged and seeded onto 25 cm^2^ culture flasks (Corning Incorporated, Corning, NY, USA) coated with 0.1% gelatin (Sigma-Aldrich, Darmstadt, Germany) and modified ‘preplate’ method was used to obtain human primary myoblast cultures. The same procedure was performed for health control. The cells were maintained at the standard in vitro culture conditions (5% CO_2_, 37 °C, 95% humidity), after reaching about 80% confluency, the cells were passaged in split ratio- 1:3 using trypsin/EDTA solution (Sigma-Aldrich, Darmstadt, Germany).

#### 4.2.2. Total RNA Extraction from Cells

The total RNA was extracted from 3 × 10^6^ cells using RNeasy Plus Mini Kit (Qiagen, Germantown, MD, USA). The quantity of isolated RNA was evaluated with a NanoDrop ND-1000 spectrophotometer (Nanodrop Technologies Inc., Wilmington, DE, USA).

#### 4.2.3. Reprogramming of Human Myoblast Cells

Myoblasts were reprogrammed with CytoTune 2.0 kit as described in the manufacturer’s protocol (Thermo Fisher Scientific, Carlsbad, CA, USA) using Sendai particles to deliver Yamanaka’s factors. After reaching about 100% confluency, the cells were passaged using 0.5 mM EDTA, at pH 8.0 solution and split in a ratio 1:10. Obtained iPS were fully characterized for gene expression-qPCR (*dystrophin*, *OCT4*, *SOX2*, *NANOG*, and *C-MYC*); and marker proteins immunofluorescence (dystrophin, OCT4, SOX2, C-MYC, TRA1-60, and SSEA4).

#### 4.2.4. The iPSc Culture

Disaggregation of pluripotent colonies to single cells was supported by ROCK inhibitors to prevent high cell mortality. The cells were seeded on Matrigel-coated dishes in appropriate density in commitment medium. The commitment medium was changed every 2–3 days. Seven days after in vitro cultivation, the cells were detached (using 0.5 mM EDTA) and seeded. Feeder-free human iPS cells were cultured in Essential 8 medium on a Geltrex matrix. For passaging, cells were detached with 0.5 mM EDTA in PBS and split in a ratio of 1:10.

#### 4.2.5. Real-Time Polymerase Chain Reaction (Real-Time PCR; qPCR)

The primer pairs for each studied gene were obtained commercially from Genomed (Kraków, Poland). The cDNA was synthesized from 3 μg of total RNA using iScript™ Reverse Transcription Supermix (Bio-Rad Laboratories, Hercules, CA, USA) in 60 μL reaction volume. The Real-time PCR was performed using ready to use Real-time PCR primer assays which consisted of unlabeled PCR primer pairs (Supplementary Table S1) with SsoAdvanced™ SYBR^®^ Green supermix (Bio-Rad Laboratories, Hercules, CA, USA) or using designed in Primer-BLAST software primers (version 2.5.0) described in Supplementary Table S2 (Genomed, Krakow, Poland). The threshold cycle(Ct) values of each studied transcript were analyzed with CFX384 Touch™ Real-time PCR detection system (Bio-Rad Laboratories, Hercules, CA, USA) using standard cycling parameters. All samples and standard curves were run in duplicates. The relative expression level of each studied transcript was normalized with reference to three housekeeping genes according to GeNorm algorithm.

#### 4.2.6. Differentiation from Pluripotent Cells to Human iPS Cell-Derived Mesoangioblast-like Cells (HIDEMs)

This part of the study was based on the protocol developed by the Maffioletti group [6]. HIDEMs were derived from human iPS cells. The stabilized cultured cell phenotypes were verified by flow cytometry and immunofluorescence using following markers: CD13, CD44, CD49b (mesoangioblast-like cells normally more than 50% positive), CD31 (endothelial cell marker), CD56 (myoblast marker), CD45 (panhematopoietic marker), CD73, CD105 (mesoangioblast-like cells are normally positive), CD146 (a vascular/perivascular marker in vivo), alkaline phospathase (AP), and SSEA4 (Abcam, Cambridge, UK) (listed in Supplementary Table S3)

#### 4.2.7. Immunofluorescence Staining

For immunofluorescence staining, passaged iPS cells and HIDEMs were transferred to a 12-well plate, with a pre-prepared round coverslip in each well. Cells were cultured until they reached a confluence of about 60–70%. The plates were then transferred to ice, the cells were washed twice in 1 mL of PBS/well (after 5 min of incubation); after washing, the cells were fixed with 4% paraformaldehyde solution and incubated for 15 min. After the incubation was over, the cells were re-washed twice with 1 mL of PBS/well (after 5 min of incubation). The fixed co-cell preparations were stored at −20 °C until an adequate number of samples was obtained. Slides covered with cells were placed in the previously prepared staining chambers, keeping the slides constantly moist. The slides were washed with 0.5 mL of PBS/5 min/slide, after removing the liquid, a solution of 0.1% Triton X-100 in PBS was applied to the slides and the cells were incubated for 15 min. Then the fluid was removed and 1% goat serum solution in 0.1% Triton X-100 solution in PBS was applied and incubated for 1 h. After incubation, the liquid was removed and a 0.5 mL solution of 0.1% Triton X-100 in PBS containing the primary antibody was applied at the dilution recommended by the manufacturer. Cells were incubated for 24 h at room temperature; after incubation, cells were washed twice using PBS. The fluid was then removed and the solution containing the secondary antibody was applied according to the manufacturer’s recommendations. The cells were incubated for 1 h, then the fluid was removed and the cells were washed twice with PBS. After removing the buffer, the slides were inverted and placed with tweezers on a primary slide. Immunofluorescence staining was carried out under light-limited conditions using a fluorescence microscope (Olympus, BX 40, Tokyo, Japan). The antibodies used have been described in Supplementary Table S3.

#### 4.2.8. Lentiviral Packaging

Lentivector packing protocol was performed as earlier described. Briefly, at day 0: the 25 × 10^6^ of HEK293T cells were plated in 15-cm plates to produce the lentivirus. At day 1: construct containing micro-dystrophin was mixed with packaging plasmid (psPAX) and envelop plasmid (MD2G) at the ratio 4:3:1 and was added to cell medium. 48 h post-transfection, the virus-containing medium was collected from each plate and filtered through a 0.45 μm filter to remove debris and floating packaging cells. In order to prepare a concentrate, the centrifugation using Millipore concentrator (Amicon, Darmstadt, Germany) 10,000× *g* for at least 1 h at 4 °C was performed. Concentrated viral particles were aliquoted, snap-frozen and stored at −80 °C. At this step, RNA virus was extracted and titer determined using Lenti-X qRT-PCR Titration Kit (Takara Bio, San Jose, CA, USA). MOI (multiplicity of infection) was verified using five different concentrations of virus to optimize the number of vector particles per cell [35].

#### 4.2.9. Lentiviral Transduction/Genetic Modification

HIDEMs were modified using the Lentiviral vector Vecotr Builder Inc., Fort Worth, TX, USA). At day 0: cells were plated at the density of 8 × 10^5^ target cells per 10-cm plate. On day 1: Lentiviral transductions were performed by mixing cells and virus in culture medium supplemented with polybrene. On day 2: 24 h post-transduction, the virus-medium was diluted using fresh medium. On day 3: the medium was replaced with fresh medium without Polybrene (or Protamine sulfate) and grown under standard in vitro conditions. On day 4–5: determination of the % of transduced (GFP-positive) cells was established by fluorescence microscope. On day 6–7: selection using antibiotic puromycin was performed [35].

#### 4.2.10. Flow Cytometry

Cells were stained with selected antibodies in the dilutions specified in manufacturer’s guidelines. Then the cells were washed with PBS containing 1% FBS and analyzed on the CytoFLEX S flow cytometer (Beckman Coulter, Indianapolis, IN, USA).

#### 4.2.11. Catalase Assay Kit

Cells were harvested due to manufacturer’s instructions (Cayman Chemicals, Catalase Assay Kit, Ann Arbor, MI, USA), then counted manually to get the dilution of 1 million cells in 1 mL of PBS. Then samples were sonicated for 2 min and kept in −80 °C freezer until the test performance. The assay was performed according to the manufacturer’s instructions.

#### 4.2.12. Superoxide Dismutase Assay Kit

Cells were harvested due to manufacturer’s instructions (Cayman Chemicals, Superoxide Dismutase Assay Kit, Ann Arbor, MI, USA), then counted manually to get the dilution of 1 million cells in 1 mL of PBS. Then samples were sonicated for 2 min and kept in −80 °C freezer until the test performance. The assay was performed according to the manufacturer’s instructions.

#### 4.2.13. Antioxidant Assay Kit

Cells were harvested due to manufacturer’s instructions (Cayman Chemicals, Antioxidant Assay Kit, Ann Arbor, MI, USA), then counted manually to get the dilution of 1 million cells in 1 mL of PBS. Then samples were sonicated for 2 min and kept in −80 °C freezer until the test performance. The assay was performed according to the manufacturer’s instructions.

#### 4.2.14. Animal Study

Animals subjected to intramuscular interventions were under oral anesthetic (a mixture of oxygen and isoflurane) and were given a single i.m. dose of cell suspension into the *tibialis anterior* muscle of both limbs (cells were resuspended in Ca^2+^-and Mg^2+^ -free PBS to a concentration of 10^6^ cells/30 μL per animal) strictly following a protocol described by Gerli et al. [21]. A procedure with physiological saline solution in the same fluid volume to identical sites was performed in the SHAM mouse group while control *mdx* mice were not given any injections.

#### 4.2.15. Treadmill Test

Mice were exercised on a treadmill once a week for five minutes at a speed of twelve meters per minute to examine their physical strength in order to confirm the possible therapeutic impact. If the animal tried to escape or became inactive, an electric shock of 1 Hz and 0.5 A was activated. Data of distance length, time and quantity of electrical impulses that the animal received during the experiment were collected. The equipment stopped the experiment when the mouse was inactive for an extended length of time or when it took several pulses in a little amount of time, protecting the mouse from harm. Experiment was performed according to Rodgers et al. [36].

#### 4.2.16. Statistical and Bioinformatical Analysis

Data were analyzed with GraphPad Prism (GraphPad Prism 5 Software, San Diego, CA, USA), using appropriate statistical tests and values obtained which were considered to be significant at *p* < 0.05. Continuous data were compared using t-tests for two independent groups and one-way ANOVA for 3 or more groups.

## Figures and Tables

**Table 1:** Descriptive statistics of the results of the distance travelled test on the treadmill of the mice test groups.

	Mice WT (n = 4)	Mice *mdx* Control (n = 4)	Mice *mdx* SHAM (n = 4)	Mice *mdx* After HIDEMs + *μDys* Administration (n = 4)	Mice *mdx* After HIDEMs Administration (n = 4)
Minimum	58	2	3	2	2
Maximum	60	60	60	60	60
Median	60	60	60	60	60
Mean	59.92	49.25	49.34	47.94	44.16
Standard Deviation +/−	0.37 +/−	21.32 +/−	19.45 +/−	21.85 +/−	23.61 +/−

**Table 2:** Descriptive statistics of the number of electric shocks inflicted to test *mdx* mice groups during treadmill exercise.

	Mice WT (n = 4)	Mice *mdx* Control (n = 4)	Mice *mdx* SHAM (n = 4)	Mice *mdx* After HIDEMs + *μDys* Administration (n = 4)	Mice *mdx* After HIDEMs Administration (n = 4)
Minimum	0	0	0	0	1
Maximum	12	100	88	40	96
Median	1	1	3	2	35
Mean	1.53	10.94	13.72	5.53	40.56
Standard Deviation	2.38	24.80	21.03	9.21	32.87

**Table 3:** The summary of patients’ information.

	Patient 34	Patient 38	Patient 39	Patient 40	Healthy Control
Age	11	12	15	8	18
Dystrophin gene mutation	Deletion of exons 8–22 (out of frame)	Deletion of exons 51–57 (in frame)	Duplication of exons 53–57	Deletion of exons 2–17 (out of frame) Deletion of exons 30–44 (in frame)	--
Disorder	De novo	Inherited	Inherited	Inherited	--
Functional status	Wheelchair from age 9	Still ambulant, needs support when walking	Tetraplegic	Tetraplegic	--

## Data Availability

Data is contained within the article.

## Supplementary Materials

The following supporting information can be downloaded at: https://www.mdpi.com/article/10.3390/ijms252211869/s1.

## References

[1] Mendell J.R., Shilling C., Leslie N.D., Flanigan K.M., Al-Dahhak R., Gastier-Foster J., Kneile K., Dunn D.M., Duval B., Aoyagi A. (2012). Evidence-Based Path to Newborn Screening for Duchenne Muscular Dystrophy. Ann. Neurol..

[2] Bushby K.M.D., Gardner-Medwin D., Nicholson L.V.B., Johnson M.A., Haggerty I.D., Cleghorn N.J., Harris J.B., Bhattacharyal S.S. (1993). The Clinical, Genetic and Dystrophin Characteristics of Becker Muscular Dystrophy—II. Correlation of Phenotype with Genetic and Protein Abnormalities. J. Neurol..

[3] Van Putten M., Hulsker M., Young C., Nadarajah V.D., Heemskerk H., Van Der Weerd L., ’T Hoen P.A.C., Van Ommen G.J.B., Aartsma-Rus A.M. (2013). Low Dystrophin Levels Increase Survival and Improve Muscle Pathology and Function in Dystrophin/Utrophin Double-Knockout Mice. FASEB J..

[4] McDonald C.M., Marbán E., Hendrix S., Hogan N., Ruckdeschel Smith R., Eagle M., Finkel R.S., Tian C., Janas J., Harmelink M.M. (2022). Repeated Intravenous Cardiosphere-Derived Cell Therapy in Late-Stage Duchenne Muscular Dystrophy (HOPE-2): A Multicentre, Randomised, Double-Blind, Placebo-Controlled, Phase 2 Trial. Lancet.

[5] Komaki H., Nagata T., Saito T., Masuda S., Takeshita E., Sasaki M., Tachimori H., Nakamura H., Aoki Y., Takeda S. (2018). Systemic Administration of the Antisense Oligonucleotide NS-065/NCNP-01 for Skipping of Exon 53 in Patients with Duchenne Muscular Dystrophy. Sci. Transl. Med..

[6] Maffioletti S.M., Gerli M.F.M., Ragazzi M., Dastidar S., Benedetti S., Loperfido M., Vandendriessche T., Chuah M.K., Tedesco F.S. (2015). Efficient Derivation and Inducible Differentiation of Expandable Skeletal Myogenic Cells from Human ES and Patient-Specific iPS Cells. Nat. Protoc..

[7] Fittipaldi S., Dimauro I., Mercatelli N., Caporossi D. (2014). Role of Exercise-Induced Reactive Oxygen Species in the Modulation of Heat Shock Protein Response. Free Radic. Res..

[8] Dudley R.W.R., Khairallah M., Mohammed S., Lands L., Des Rosiers C., Petrof B.J. (2006). Dynamic Responses of the Glutathione System to Acute Oxidative Stress in Dystrophic Mouse (Mdx) Muscles. Am. J. Physiol. Regul. Integr. Comp. Physiol..

[9] Kaczor J.J., Hall J.E., Payne E., Tarnopolsky M.A. (2007). Low Intensity Training Decreases Markers of Oxidative Stress in Skeletal Muscle of Mdx Mice. Free Radic. Biol. Med..

[10] Renjini R., Gayathri N., Nalini A., Bharath M.M.S. (2012). Oxidative Damage in Muscular Dystrophy Correlates with the Severity of the Pathology: Role of Glutathione Metabolism. Neurochem. Res..

[11] Tidball J.G. (2005). Inflammatory Processes in Muscle Injury and Repair. Am. J. Physiol. Regul. Integr. Comp. Physiol..

[12] Barbieri E., Sestili P. (2012). Reactive Oxygen Species in Skeletal Muscle Signaling. J. Signal Transduct..

[13] Lamon S., Wallace M.A., Russell A.P. (2014). The STARS Signaling Pathway: A key Regulator of Skeletal Muscle Function. Pflug. Arch. Eur. J. Physiol..

[14] Lamon S., Wallace M.A., Léger B., Russell A.P. (2009). Regulation of STARS and Its Downstream Targets Suggest a Novel Pathway Involved in Human Skeletal Muscle Hypertrophy and Atrophy. J. Physiol..

[15] Chai J., Tarnawski A.S. (2002). Serum Response Factor: Discovery, Biochemistry, Biological Roles and Implications for Tissue Injury Healing. J. Physiol. Pharmacol..

[16] Hill C.S., Wynne J., Treisman R. (1995). The Rho Family GTPases RhoA, Racl, and CDC42Hsregulate Transcriptional Activation by SRF. Cell.

[17] Sotiropoulos A., Gineitis D., Copeland J., Treisman R. (1999). Signal-Regulated Activation of Serum Response Factor is Mediated by Changes in Actin Dynamics. Cell.

[18] Miralles F., Posern G., Zaromytidou A.I., Treisman R. (2003). Actin Dynamics Control SRF Activity by Regulation of its Coactivator MAL. Cell.

[19] Sisson T.H., Ajayi I.O., Subbotina N., Dodi A.E., Rodansky E.S., Chibucos L.N., Kim K.K., Keshamouni V.G., White E.S., Zhou Y. (2015). Inhibition of Myocardin-Related Transcription Factor/Serum Response Factor Signaling Decreases Lung Fibrosis and Promotes Mesenchymal Cell Apoptosis. Am. J. Pathol..

[20] Sandbo N., Smolyaninova L.V., Orlov S.N., Dulin N.O. (2016). Control of Myofibroblast Differentiation and Function by Cytoskeletal Signaling. Biochemistry.

[21] Gerli M.F., Maffioletti S.M., Millet Q., Tedesco F.S. (2014). Transplantation of induced pluripotent stem cell-derived mesoangioblast-like myogenic progenitors in mouse models of muscle regeneration. J. Vis. Exp..

[22] Potter R.A., Griffin D.A., Heller K.N., Peterson E.L., Clark E.K., Mendell J.R., Rodino-Klapac L.R. (2021). Dose-Escalation Study of Systemically Delivered rAAVrh74.MHCK7.Micro-Dystrophin in the Mdx Mouse Model of Duchenne Muscular Dystrophy. Hum. Gene Ther..

[23] Mendell J.R., Sahenk Z., Lehman K., Nease C., Lowes L.P., Miller N.F., Iammarino M.A., Alfano L.N., Nicholl A., Al-Zaidy S. (2020). Assessment of Systemic Delivery of rAAVrh74.MHCK7.Micro-Dystrophin in Children with Duchenne Muscular Dystrophy: A Nonrandomized Controlled Trial. JAMA Neurol..

[24] Périé S., Trollet C., Mouly V., Vanneaux V., Mamchaoui K., Bouazza B., Marolleau J.P., Laforêt P., Chapon F., Eymard B. (2014). Autologous Myoblast Transplantation for Oculopharyngeal Muscular Dystrophy: A Phase I/IIa Clinical Study. Mol. Ther..

[25] Cossu G., Previtali S.C., Napolitano S., Cicalese M.P., Tedesco F.S., Nicastro F., Noviello M., Roostalu U., Natali Sora M.G., Scarlato M. (2015). Intra-arterial Transplantation of HLA -matched Donor Mesoangioblasts in Duchenne Muscular Dystrophy. EMBO Mol. Med..

[26] Austin L., de Niese M., McGregor A., Arthur H., Gurusinghe A., Gould M.K. (1992). Potential Oxyradical Damage and Energy Status in Individual Muscle Fibres from Degenerating Muscle Diseases. Neuromuscul. Disord..

[27] Hauser E., Hoger H., Bittner R., Widhalm K., Herkner K., Lubec G. (1995). Oxyradical Damage and Mitochondrial Enzyme Activities in the Mdx Mouse. Neuropediatrics.

[28] Aaron Hobbs G., Zhou B., Cox A.D., Campbell S.L. (2014). Rho GTPases, Oxidation, and Cell Redox Control. Small GTPases.

[29] Kim J.G., Choi K.C., Hong C.W., Park H.S., Choi E.K., Kim Y.S., Park J.B. (2017). Tyr42 Phosphorylation of RhoA GTPase Promotes Tumorigenesis Through Nuclear Factor (NF)-κB. Free Radic. Biol. Med..

[30] Kim J.G., Kim M.J., Choi W.J., Moon M.Y., Kim H.J., Lee J.Y., Kim J., Kim S.C., Kang S.G., Seo G.Y. (2017). Wnt3A Induces GSK-3β Phosphorylation and β-Catenin Accumulation Through RhoA/ROCK. J. Cell. Physiol..

[31] Li Y.P. (2003). TNF-α is a Mitogen in Skeletal Muscle. Am. J. Physiol. Cell Physiol..

[32] Sadler K.J., Gatta P.A.D., Naim T., Wallace M.A., Lee A., Zaw T., Lindsay A., Chung R.S., Bello L., Pegoraro E. (2021). Striated Muscle Activator of Rho Signalling (STARS) Overexpression in the Mdx Mouse Enhances Muscle Functional Capacity and Regulates the Actin Cytoskeleton and Oxidative Phosphorylation Pathways. Exp. Physiol..

[33] Mendell J.R., Al-Zaidy S., Shell R., Arnold W.D., Rodino-Klapac L.R., Prior T.W., Lowes L., Alfano L., Berry K., Church K. (2017). Single-Dose Gene-Replacement Therapy for Spinal Muscular Atrophy. N. Engl. J. Med..

[34] Lowes L.P., Alfano L.N., Arnold W.D., Shell R., Prior T.W., McColly M., Lehman K.J., Church K., Sproule D.M., Nagendran S. (2019). Impact of Age and Motor Function in a Phase 1/2A Study of Infants With SMA Type 1 Receiving Single-Dose Gene Replacement Therapy. Pediatr. Neurol..

[35] Fiedorowicz K., Rozwadowska N., Zimna A., Malcher A., Tutak K., Szczerbal I., Nowicka-Bauer K., Nowaczyk M., Kolanowski T.J., Łabędź W. (2020). Tissue-specific Promoter-based Reporter System for Monitoring Cell Differentiation from iPSCs to Cardiomyocytes. Sci. Rep..

[36] Rodgers B.D., Bishaw Y., Kagel D., Ramos J.N., Maricelli J.W. (2020). Micro-dystrophin Gene Therapy Partially Enhances Exercise Capacity in Older Adult Mdx Mice. Mol. Ther. Methods Clin. Dev..

