# Supplementary material for: In Vitro Gene Therapy Using Human iPS-Derived Mesoangioblast-Like Cells (HIDEMs) Combined with Microdystrophin (μDys) Expression as the New Strategy for Duchenne Muscular Dystrophy (DMD) Experimental Treatment
Source: Int J Mol Sci. 2024 Nov 5;25(22):11869. doi: 10.3390/ijms252211869 (PMC11593506; doi:10.3390/ijms252211869)
Supplement: Supplementary file 1 [file ijms-25-11869-s001.zip › ijms-3170015-supplementary.pdf]

Supplementary Table S1. List of primers for PCR products for identifying genes studied in tested cell suspensions as provided by BioRad Laboratories (manufacturer restricted the information concerning primer sequences).

Target Gene	Name of the BioRad product
ANPEP (CD 13)	PrimePCR SYBR Green Assay: ANPEP, Human
CD 44 (CD 44)	PrimePCR SYBR Green Assay: CD 44, Human
ITGA2 (CD 49b)	PrimePCR SYBR Green Assay: ITGA2, Human
MCAM (CD 146)	PrimePCR SYBR Green Assay: MCAM, Human
NT5E (CD 73)	PrimePCR SYBR Green Assay: NT5E, Human
PTPRC (CD 45)	PrimePCR SYBR Green Assay: PTPRC, Human
ACTB	PrimePCR SYBR Green Assay: ACTB, Human
GAPDH	PrimePCR SYBR Green Assay: GAPDH, Human
HPRT 1	PrimePCR SYBR Green Assay: HPRT1, Human
POU5F1 (OCT4)	PrimePCR SYBR Green Assay: POU5F1, Human
SOX2	PrimePCR SYBR Green Assay: SOX2, Human
NANOG	PrimePCR SYBR Green Assay: NANOG, Human
MINA (C-MYC)	PrimePCR SYBR Green Assay: MINA, Human
GAPDH	PrimePCR SYBR Green Assay: GAPDH, Human
TBP	PrimePCR SYBR Green Assay: TBP, Human

Supplementary Table S2. List of primers and expected length of the PCR product to genes of Rho signaling pathway and *μDYS* sequence designed in Primer-BLAST software, Genomed, Warsaw, Poland.

Target Gene	Sequence (5'->3')	Length of PCR product
STARS	Forward: CAGGTACGAGAGGGATGCTG	110 nt
	Reverse: GGTTGGCACATTTTCTCCTCC	
MRFTA2	Forward: GAAGAGAGCCAGACTAGCCG	132 nt
	Reverse: TTCACCTGGCCCACAATGATG	
RHO	Forward: TCTGTCCCAACGTGCCCATCAT	118 nt
	Reverse: CTGCCTTCTTCAGGTTTCACCG	
NF-kB	Forward: GCAGCACTACTTCTTGACCACC	130 nt
	Reverse: TCTGCTCCTGAGCATTGACGTC	
TNF-a	Forward: CCAGGCAGTCAGATCATCTTCTC	144 nt
	Reverse: TTATCTCTCAGCTCCACGCCA	
SRF	Forward: CCTCAACTCGCCAGACTCTC	143 nt
	Reverse: CCGGCTTCAGTGTGTCCTTG	
μDYS	Forward: TCAGCAGAAAGAAGCCACGA	172 nt
	Reverse: AGGCTGCTGCTTATGTCACCACC	

Supplementary Table S3. Antibody characteristics

A The list of antibodies used for immunofluorescent staining.

Marker	Target Antigen	Antibody
Mesenchymal-like cell characteristics<br><br>Characteristic for HIDEMs	AP	alkaline phospathase, ab126820, Abcam, Great Britain
	CD 13	Anti-CD 13, ab227663, Abcam, Great Britain
	CD 31	Anit-CD 31, ab9498, Abcam, Great Britain
	CD 44	Anit-CD 44, ab189524, Abcam, Great Britain
	CD 45	Anit-CD 45, ab821, Abcam, Great Britain
	CD 73	Anit-CD 73, ab133582, Abcam, Great Britain
	CD105	Anit-CD 105, ab11414, Abcam, Great Britain
	CD 146	Anit-CD 146, ab75769, Abcam, Great Britain
Pluripotency markers<br><br>Characteristic for iPS	C-MYC	Anit-c-Myc ab32072, Abcam, Great Britain
	NANOG	Anit-Nanog, ab21624, Abcam, Great Britain
	OCT4	Anit-Oct4, ab19857, Abcam, Great Britain
	SOX2	Anit-SOX2, ab97959, Abcam, Great Britain
	SSEA4	Anit-SSEA4, ab16287, Abcam, Great Britain
	TRA1-60	Anit-TRA-1-60, ab16288, Abcam, Great Britain
Fluorescence Secondary antibodies	Ms AlexaFluor 488	ab130113, Abcam, Great Britain
	Rb AlexaFluor 594	ab150076, Abcam, Great Britain

B The list of antibodies used in flow cytometry.

Marker	Target antigen	Antibody
Mesenchymal-like cell characteristics<br><br>(HIDEMs)	AP	PE Mouse anti-Human Alkaline Phosphatase, 561433, BD Pharmigen, USA
	CD 13	APC Mouse Anti-Human CD13, 557454, BD Pharmigen, USA
	CD 31	PE Mouse Anti-Human CD31, 555446, BD Pharmingen, USA
	CD 44	FITC Mouse Anti-Human CD44, 555478, BD Pharmingen, USA
	CD 45	APC Mouse Anti-Human CD45, 555485, BD Pharmingen, USA
	CD 49b	FITC Mouse Anti-Human CD49b, 555498, BD Pharmigen, USA

	CD 56	CD56-PC5, A07789, Beckman Coulter, USA
	CD 73	APC Mouse anti-Human CD73, 560847, BD Pharmingen, USA
	CD105	PE Mouse anti-Human CD105, 560839, BD Pharmingen, USA
	CD 146	PE Mouse Anti-Human CD146, 550315) BD Pharmingen, USA
Control antibodies	IgG1 Isotype control	FITC Mouse IgG1 Isotype control, 555748, BD Pharmigen, USA
	IgG1 Mouse-PC5	IgG1 Mouse-PC5, A07798, Beckman Coulter, USA
	IgG2b κ Isotype Control	FITC Mouse IgG2b κ Isotype Control, 555742, BD Pharmingen, USA
	IgG1, κ Isotype Control	APC Mouse IgG1, κ Isotype Control, 555751 BD Pharmingen, USA
	IgG1, κ Isotype Control	PE Mouse IgG1, κ Isotype Control, 554680, BD Pharmingen, USA
